# Case Report: Early-onset Luminal B-like male breast cancer with perineural invasion

**DOI:** 10.3389/fonc.2026.1968604

**Published:** 2026-10-13

**Authors:** Limin Fan, Lin Tan, Wu Tang

**Affiliations:** Department of General Surgery, Guangzhou Twelfth People’s Hospital, Guangzhou, China

**Keywords:** early-stage breast cancer, endocrine therapy, Luminal B subtype, male breast cancer, modified radical mastectomy, perineural invasion

## Abstract

Male breast cancer (MBC) is a rare malignancy accounting for less than 1% of all breast cancers and less than 1% of cancers in men. Compared with female breast cancer, MBC is characterized by a predominance of hormone receptor-positive luminal tumors, whereas evidence guiding individualized management remains largely extrapolated from female populations. Perineural invasion (PNI), a recognized adverse prognostic factor in several solid tumors, is uncommon in breast cancer and has rarely been reported in men. Its clinicopathological significance and therapeutic implications in male breast cancer remain poorly defined. We report a young man with stage IA Luminal B-like (HER2-negative) MBC complicated by PNI to highlight the challenges of postoperative risk stratification and adjuvant treatment decision-making.

## Introduction

Male breast cancer (MBC) is a rare malignancy, accounting for approximately 1% of all breast cancers and less than 1% of cancers diagnosed in men (1). Although MBC remains uncommon, population-based studies suggest a modest increase in incidence over recent decades (2–4). The median age at diagnosis is approximately 67 years, which is 5–10 years older than that of female breast cancer (FBC), and patients typically present with a painless retroareolar mass (5, 6). Compared with FBC, MBC exhibits distinct clinicopathological characteristics, including a marked predominance of hormone receptor-positive (HR+) luminal subtypes, with more than 90% of tumors expressing estrogen and/or progesterone receptors (7).

Despite these biological differences, clinical management of MBC has historically been extrapolated from evidence derived primarily from FBC due to the scarcity of prospective male-specific clinical trials (8). Younger-onset MBC represents an uncommon subgroup and is frequently associated with hereditary cancer susceptibility, particularly germline pathogenic variants in BRCA2. However, the biological characteristics and optimal risk stratification strategies for young male patients remain incompletely defined.

Perineural invasion (PNI), characterized by tumor cell infiltration within, around, or through the nerve sheath, is an established adverse prognostic factor in several malignancies (9, 10). In breast cancer, emerging evidence suggests that PNI is associated with aggressive pathological features and inferior clinical outcomes; however, existing data are predominantly derived from FBC cohorts. Given the rarity of MBC, the prevalence, prognostic significance, and clinical implications of PNI in male patients remain poorly understood (11).

Herein, we report a rare case of a 49-year-old man with stage IA Luminal B-like (HER2-negative) MBC accompanied by PNI who underwent total mastectomy and sentinel lymph node biopsy with postoperative adjuvant systemic therapy. Through a comprehensive review of the current literature, we discuss the clinicopathological characteristics of MBC, the potential prognostic significance of PNI, and the challenges of individualized treatment decision-making in this rare patient population.

## Case presentation

A 49-year-old Chinese man presented with a painless left retroareolar breast mass that had been present for approximately three years and had progressively enlarged during the preceding six months. On physical examination, the mass was located at the 6 o’clock position beneath the left nipple, measuring approximately 2 × 1.5 cm. It was firm in consistency with relatively well-defined margins and good mobility, showing no fixation to the overlying skin or underlying pectoral muscles. There was no nipple retraction, nipple discharge, or skin changes. No enlarged lymph nodes were palpable in the bilateral axillae or supraclavicular regions. The patient had no significant comorbidities or previous history of breast or other malignancies and was not taking any regular medications. He had a documented allergy to levofloxacin. He had no history of smoking or alcohol consumption. There was no known family history of breast or ovarian cancer.

Breast ultrasonography demonstrated a hypoechoic lesion in the left retroareolar region measuring approximately 21 × 14 × 13mm, characterized by irregular margins and internal vascularity (**Figure 1A**). Given the suspicious imaging findings, an initial excisional biopsy was performed for definitive diagnosis. Histopathological examination revealed invasive carcinoma of breast origin. Immunohistochemical evaluation demonstrated diffuse and strong expression of estrogen receptor (ER, 98%; Allred score 8) and progesterone receptor (PR, 95%; Allred score 8), while human epidermal growth factor receptor 2 (HER2) was negative (immunohistochemistry score 0). The Ki-67 proliferation index was 20%+ (**Figure 2**). The tumor was classified as Luminal B-like according to the 2013 St. Gallen Consensus and the CSCO Guidelines for Breast Cancer Diagnosis and Treatment (2026 Edition) (12, 13).

**Figure 1:**
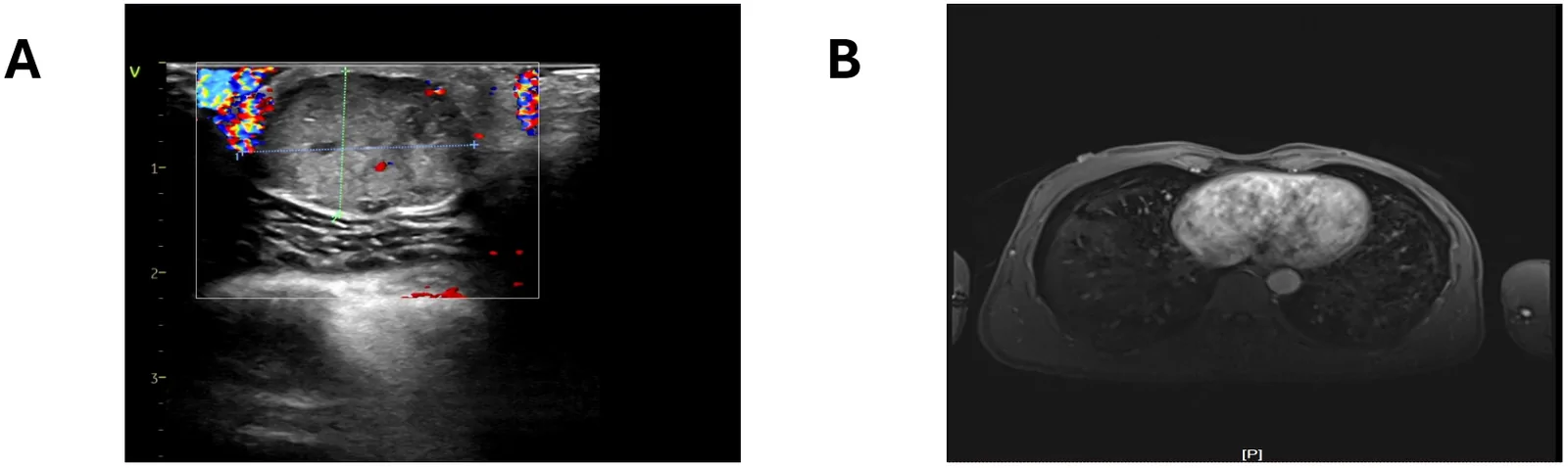
**(A)** Preoperative color Doppler ultrasound. **(B)** Postoperative MRI follow-up six months later.

**Figure 2:**
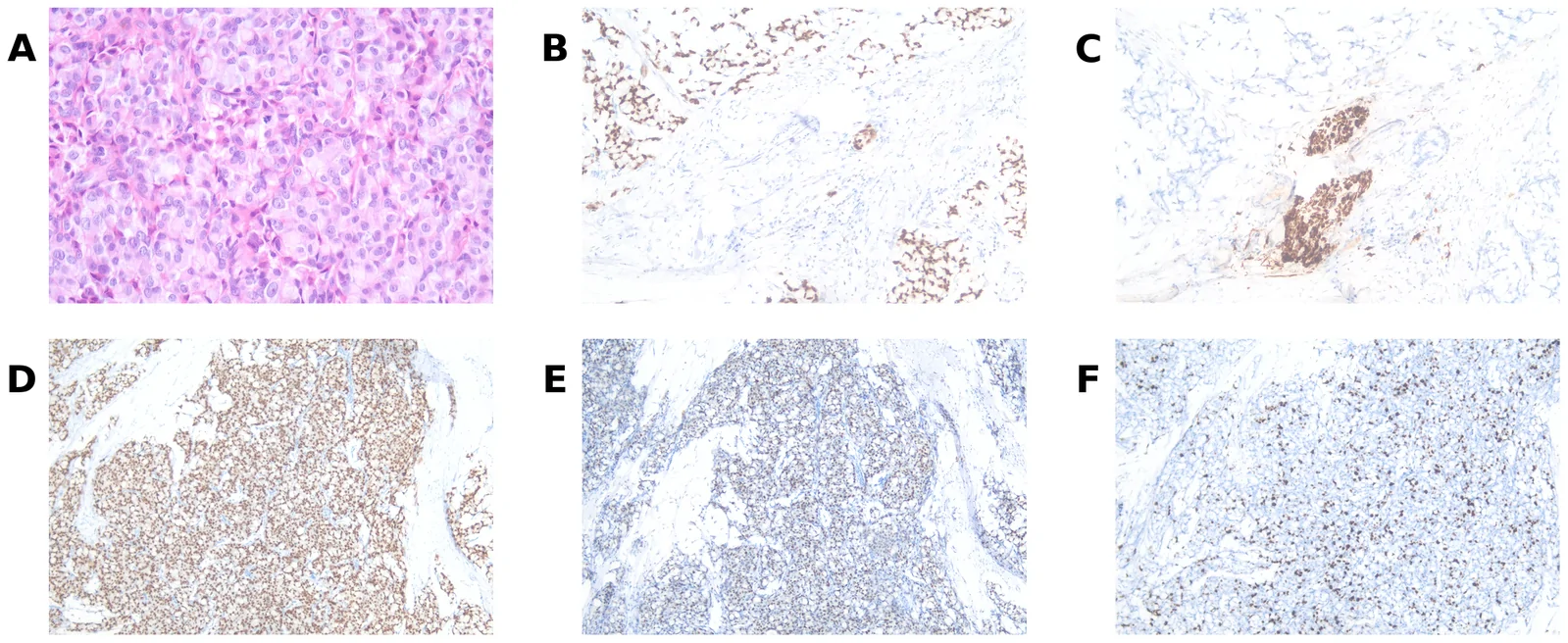
**(A)** Hematoxylin-eosin staining of breast tumor biopsy specimens. HE staining and immunohistochemistry results confirming the diagnosis of invasive carcinoma. **(B)** GATA3(+). **(C)** S-100(+). **(D)** ER (+). **(E)** PR (+). **(F)** Ki-67(20+).

Subsequent contrast-enhanced breast MRI was performed for local staging and treatment planning. No additional multifocal lesions, suspicious chest wall involvement, or radiologically abnormal axillary lymph nodes were identified. The patient subsequently underwent left total mastectomy with sentinel lymph node biopsy. Final pathological examination confirmed invasive carcinoma of no special type (NST), Nottingham histological grade III (total score 8; tubule formation 3, nuclear pleomorphism 3, mitotic count 2). Perineural invasion (PNI) was identified (**Figure 2**), whereas lymphovascular invasion was absent. All four sentinel lymph nodes were negative for metastatic carcinoma (0/4), and surgical margins were free of tumor involvement. According to the American Joint Committee on Cancer (AJCC) 8th edition staging system, the tumor was classified as pT1cN0M0, corresponding to stage IA disease.

The postoperative recovery was uneventful, and the patient was discharged without surgical complications. Despite the presence of several potentially unfavorable biological features, including young age, high histological grade, Luminal B-like phenotype, and PNI, the patient has been taking tamoxifen continuously since the surgery. At the six-month follow-up, there was no clinical or radiological evidence of recurrence. (**Figure 1B**). Contrast-enhanced breast MRI demonstrated no suspicious recurrent lesions, and serum breast cancer-related tumor markers remained within normal limits. The treatment timeline was shown in **Figure 3**.

**Figure 3:**
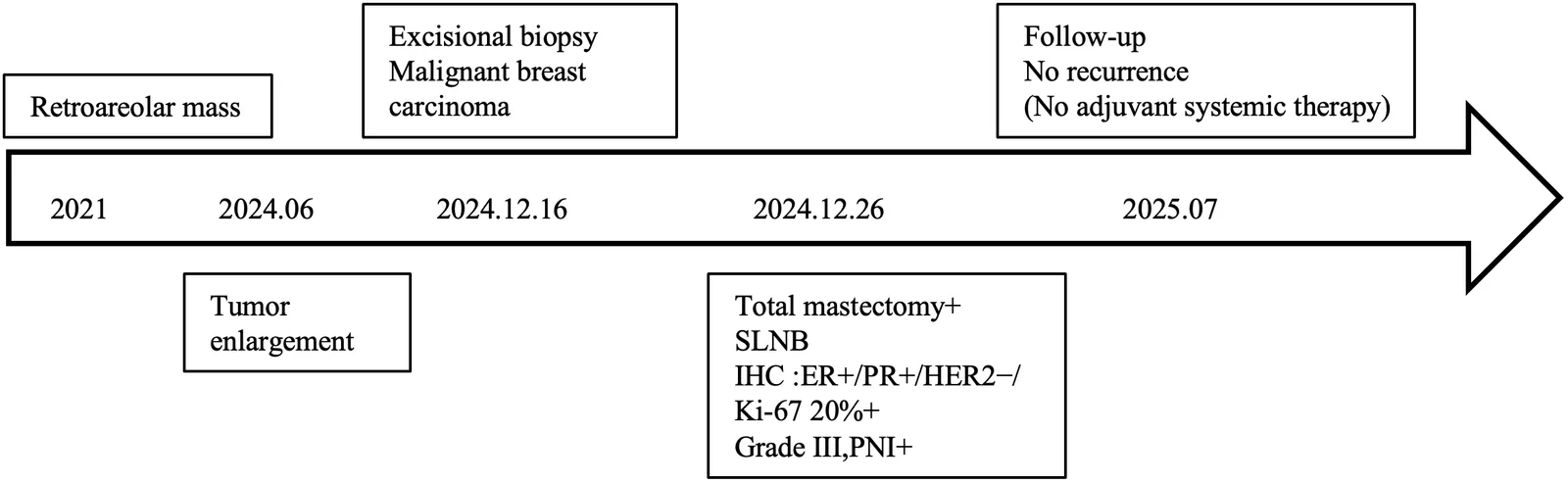
Timeline of treatments.

## Discussion

MBC is a rare but biologically distinct malignancy, accounting for less than 1% of all breast cancers (1). Unlike FBC, MBC predominantly occurs in older men and is characterized by a strong hormone receptor dependency, with more than 90% of tumors expressing estrogen receptor (ER) and/or progesterone receptor (PR) (14–17). The largest international cohort, the EORTC 10085/TBCRC/BIG/NABCG International Male Breast Cancer Program, demonstrated that MBC differs from FBC not only epidemiologically but also at the molecular level, with a marked predominance of luminal subtypes and relative scarcity of HER2-positive and triple-negative phenotypes (7). In addition, invasive carcinoma of no special type (NST) represents the predominant histological subtype in MBC, consistent with the pathological findings in our patient.

Although most MBC cases are diagnosed in elderly men, early-onset MBC represents an uncommon clinical scenario with unique biological and genetic considerations. Young male patients are more likely to harbor germline pathogenic variants, particularly in BRCA2 (18), and current guidelines recommend germline multigene panel testing for all men diagnosed with breast cancer regardless of age or family history (6). Beyond genetic susceptibility, younger patients may present with more aggressive clinicopathological features, including higher histological grade and increased proliferative activity. However, the prognostic impact of young age itself remains uncertain, as available evidence is largely derived from retrospective studies with limited sample sizes. Importantly, chronological age alone may not fully capture individual recurrence risk, and comprehensive assessment incorporating tumor biology and pathological characteristics is essential.

The present case highlights the complexity of risk stratification in young MBC. Although the patient was only 49 years old and had favorable anatomical staging (pT1cN0M0, AJCC stage IA), several adverse biological features were identified, including Nottingham grade III histology, a Luminal B-like phenotype, and PNI. This combination is uncommon in MBC and illustrates that favorable anatomical stage does not necessarily equate to low biological risk.

The Luminal B-like phenotype observed in our patient represents an important but insufficiently characterized subtype in MBC. According to the 2013 St. Gallen surrogate classification and the 2026 CSCO Breast Cancer Guidelines, HR-positive/HER2-negative tumors with high proliferative activity may be classified as Luminal B-like. In our patient, ER was 98%, PR was 95%, HER2 was 0, and the Ki-67 proliferation index was reported as 20%+, supporting the classification of the tumor as Luminal B-like. The Nottingham grade III represented an additional adverse pathological feature rather than a formal criterion for Luminal B classification. Compared with Luminal A tumors, Luminal B tumors generally exhibit greater proliferative activity and may represent a subgroup requiring more individualized risk assessment (19). However, unlike FBC, large-scale prospective studies specifically evaluating molecular subtype-based treatment strategies in MBC are lacking. Therefore, therapeutic decisions in Luminal B MBC are currently extrapolated from female breast cancer evidence and guided by clinicopathological risk factors.

PNI was another notable pathological feature in this case. PNI is defined as the presence of tumor cells within, around, or through the nerve sheath and is an established adverse prognostic factor in several solid malignancies, including pancreatic, colorectal, prostate, and head and neck cancers (20). In breast cancer, the clinical significance of PNI has only recently been recognized. Several retrospective studies in female breast cancer have demonstrated that PNI is associated with aggressive pathological characteristics, including higher histological grade, lymphovascular invasion, lymph node involvement, and inferior disease-free survival (DFS) (21–23). In this case, the coexistence of PNI, Luminal B-like phenotype, and young age suggests that anatomical stage alone may not fully capture the biological heterogeneity of MBC. Although PNI is not currently incorporated into standard adjuvant treatment algorithms, this case emphasizes its potential value as an additional pathological parameter for individualized risk stratification. Further studies specifically evaluating PNI prevalence and prognostic significance in MBC are warranted. Nevertheless, the significance of PNI in MBC remains largely unknown because most MBC cohorts lack systematic pathological evaluation of this parameter. Given the rarity of MBC, it is difficult to determine whether the prognostic impact of PNI in men parallels that observed in women.

The role of surgery alone in early-stage HR-positive MBC remains an important clinical consideration. Although mastectomy with sentinel lymph node biopsy provides excellent local control for clinically node-negative MBC, surgery alone does not address the risk of systemic recurrence associated with hormone receptor-positive disease. Evidence from female breast cancer demonstrates that endocrine therapy significantly reduces recurrence and breast cancer mortality in ER-positive tumors, with benefits extending beyond the initial five years after diagnosis (24). Similarly, retrospective MBC studies have suggested a survival advantage associated with adjuvant tamoxifen use (25). The ASCO guideline therefore recommends adjuvant tamoxifen for men with hormone receptor-positive breast cancer, with consideration of extended therapy in patients with higher-risk features (12). Postoperatively, the patient received endocrine therapy with tamoxifen in accordance with CSCO guidelines; as of the telephone follow-up in August 2026, the patient was continuing the medication.

Several limitations should be acknowledged. First, the diagnosis was initially challenging, and the patient underwent excisional biopsy of breast mass before malignancy was confirmed histologically, followed by left total mastectomy and sentinel lymph node biopsy. This sequential pathway highlights the diagnostic complexity of rare male breast cancer and the importance of timely pathological assessment and multidisciplinary management. However, we cannot determine whether this initial diagnostic procedure or the interval between procedures affected tumor biology or outcome. Second, at six months after surgery, there was no clinical or radiological evidence of recurrence, this short follow-up period cannot reliably predict long-term outcomes, particularly given the prolonged recurrence risk associated with hormone receptor-positive breast cancer. Finally, germline genetic testing was not performed, and the prognostic significance of perineural invasion in male breast cancer remains uncertain because of limited male-specific evidence. This case highlights a clinically relevant challenge in MBC management: favorable anatomical staging may underestimate biological risk, and treatment decisions should incorporate tumor characteristics beyond tumor size and lymph node status.

Adherence to endocrine therapy represents another important issue in MBC. Tamoxifen remains the preferred endocrine therapy for male patients because aromatase inhibitor monotherapy is insufficient due to persistent testicular estrogen production and incomplete estrogen suppression. However, real-world studies have demonstrated suboptimal initiation and persistence of tamoxifen therapy among men, partly due to adverse effects such as sexual dysfunction, hot flashes, mood changes, and concerns regarding masculinity and quality of life (26–28). Therefore, shared decision-making, appropriate counseling regarding treatment benefits and adverse effects, and long-term follow-up are essential components of MBC care.

Genetic counseling and surveillance are also particularly relevant in young MBC. Germline testing may identify clinically actionable variants that influence treatment selection, surveillance strategies, and familial risk assessment. In our patient, genetic testing was recommended but has not yet been completed, representing an important gap in comprehensive cancer management. Identification of hereditary susceptibility could provide valuable information regarding future cancer risks, including contralateral breast cancer and associated malignancies, while enabling cascade testing for family members.

Taken together, this case illustrates the complexity of managing young-onset MBC with high-risk biological characteristics. Although the patient presented with stage IA disease, the combination of young age, Luminal B-like phenotype, high histological grade, and PNI positivity suggest that anatomical staging alone may be insufficient for accurately estimating recurrence risk. Further studies incorporating molecular characteristics and pathological biomarkers are needed to establish more precise risk stratification models for MBC. Meanwhile, multidisciplinary evaluation, genetic counseling, and individualized systemic therapy remain essential to optimize outcomes in this rare patient population.

## Patient perspective

The patient initially experienced anxiety and uncertainty after being diagnosed with breast cancer, particularly because breast cancer is uncommon among men and relevant information regarding male-specific treatment strategies was limited. After surgical treatment, he was informed about the potential benefits and adverse effects of adjuvant systemic therapy, including endocrine therapy. Following discussion of the available evidence, potential recurrence risks, and the importance of long-term surveillance, the patient elected to initiate tamoxifen therapy and has continued treatment. At the six-month follow-up, there was no clinical or radiological evidence of recurrence. This experience highlights the importance of shared decision-making, individualized communication, and ongoing patient engagement throughout the management of rare malignancies.

## Conclusion

This case emphasizes that early-stage male breast cancer with adverse biological characteristics may not be adequately characterized by anatomical staging alone. Young age, Luminal B-like phenotype, and perineural invasion should be considered in individualized risk assessment and treatment planning. Further collaborative studies are required to clarify prognostic biomarkers and optimize adjuvant treatment strategies in this rare disease population.

## Data Availability

The original contributions presented in the study are included in the article/supplementary material. Further inquiries can be directed to the corresponding author.
